# Does player age influence match physical performance? A longitudinal four-season analysis in Spanish Soccer LaLiga

**DOI:** 10.5114/biolsport.2023.124844

**Published:** 2023-03-21

**Authors:** Tomás García-Calvo, Florentino Huertas, José Carlos Ponce-Bordón, Roberto López del Campo, Ricardo Resta, Rafael Ballester

**Affiliations:** 1 Faculty of Sport Sciences, University of Extremadura, Spain; 2 Faculty of Physical Education & Sport Sciences, Catholic University of Valencia, Spain; 3 LaLiga Sport Research Section, Madrid, Spain

**Keywords:** Age group, Aging, Football, Longitudinal analysis, Match running performance

## Abstract

This study aims to analyse the evolution of match running performance in relation to the age distribution of professional soccer players using a large-scale analysis. An explorational-longitudinal and retrospective study was designed and a total of 36,883 individual match observations were collected on outfield players competing across four consecutive Spanish LaLiga seasons (from 2015/16 to 2018/19), using an optical tracking system (ChyronHego). Soccer players were divided into 3 age groups: young (18–24 years old), middle-aged (25–30 years old), and seniors (31–41 years old). Relative total distance (TD/min), distance covered at 21–24 km · h^−1^ (HIRD/min), and > 24 km · h^−1^ per minute (VHIRD/min) were analysed; also, the number of efforts at 21–24 km · h^−1^ (Sp21) and > 24 km · h^−1^ (Sp24) were taken into consideration. Seasons were divided into four phases (P): P1 (matches 1–10), P2 (11–19), P3 (20–29), and P4 (30–38). The results showed that young players covered significantly greater TD, HIRD and VHIRD than the rest of the players (p < .05) in all season phases. In addition, TD significantly decreased along season phases in all player age group (p < .01). Crucially, young players performed significantly greater numbers of Sp21 and Sp24 than the rest of the players (p < .05) in all season phases. In addition, Sp21 and SP24 significantly decreased in middle-aged (p < .01) and senior players (p < .05) across the seasons. This study demonstrated that players’ match running performance decreases with increasing years, especially in high-intensity running distances.

## INTRODUCTION

Soccer players’ external load during competition has been widely studied [1]. External load, mainly explored through the analysis of match running performance, has been linked to different contextual-related variables such as the competitive standard and the country of the competition (First Spanish Division vs. Second Spanish Division: [2]; FA Premier League, Championship, vs. League One: [3]; three competitive leagues in Norway: [4]). Playing formation [5], playing style [6] and playing position [6, 7] have also been shown to impact running performance by means of the interplay between physical and tactical demands. Other contextual-related variables such as fixture congestion [8], match location, and match status [9], or even the recent pandemic lockdown [10], have also been related to soccer players’ external load.

The influence of age is another variable related to the extensive body of research analysing match running performance [11]. It is well known that the aging process influences athletes’ physical and competitive performance [12], but there is slight evidence from examining the effects of age on competitive match performance in professional soccer players. For instance, a study that analysed match running performance of Spanish First and Second division players in the 2017/18 season, using a computerized tracking system, reported that professional soccer players aged ≥ 30 years exhibited less total distance (TD) covered and reduced distance covered at different velocities (medium-speed: 14–21 km · h^−1^; high-speed running [HSR]: 21 km · h^−1^; very HSR: 21–24 km · h^−1^, sprinting speed: > 24 km · h^−1^) compared with younger players (< 30 years) [13]. Similarly, a recent study focused on the same season reported that players under 25.2 years showed lower variability for high-intensity activities (21–24 km · h^−1^ and > 24 km · h^−1^) in comparison with players over 33.1 years [14]. In addition, Lorenzo-Martínez et al. [15] found that soccer players aged between 31 and 38 years who competed in the First Spanish Division performed a significantly lower total number of accelerations and decelerations in comparison with younger players. However, these types of research have only considered a transversal perspective and they have not analysed the influence of age on match running performance across the years.

Considering a longitudinal perspective, recent research has shown that in elite soccer players the TD and high-intensity running distance significantly decreased each year that they got older [16]. However, this research only analysed TD and high-intensity running distance, necessitating a deeper analysis about match physical demands. In the same vein, Errekagori et al. [11] analysed the last eight seasons (from 2011–12 to 2018–19) in the Spanish Football First Division, reporting a descending trend in TD covered by soccer teams, although only TD was considered as a physical variable and age groups were not analysed. A study based on the Chinese Soccer Super League reported a significant decrease in the running performance of players, especially for high-intensity running [17]. Another similar study focused on the first German league (Bundesliga) during three seasons (2012/13–2015/16) evaluated the effects of age in match running performance, showing a significantly lower performance in professional soccer players aged > 30 years in the TD covered, the number of fast runs, and the number of sprints compared with younger players (≤ 30 years; [18]). Taken together, these results along with previous research point to a significant decrease in physical performance observed beyond the third decade of life in athletic populations [19–21]. However, the physical performance evolution across the season phases considering different age groups has not been analysed and a deeper analysis based on high-intensity running distance is also necessary. This fact is highly relevant taking into consideration that the current soccer game is evolving towards shorter, higher intensity play periods where players cover higher sprinting distances and perform more sprints and explosive actions [22–24], placing subsequently higher physical demands on the players over the temporal course of the season [25].

Thus, we consider of special interest the study of the evolution of match running performance within the season in relation to the age distribution of the players. It would also be interesting to know how physical performance changes over the years, since there are fewer studies carried out on the age effect over the seasons and these studies have only focused on analysing differences between age groups, without considering the evolution within each age group. In order to accomplish these goals, we conducted a large-scale analysis of match running performance of soccer players who competed in the First Spanish soccer league (LaLiga) across four consecutive seasons (from 2015/16 to 2018/19), also analysing the evolution of physical performance across the different phases of each season.

## MATERIALS AND METHODS

### Sample and procedure

The sample was composed of 36,883 individual match observations of 1,037 professional soccer players who competed in the First Division of the Spanish soccer league (LaLiga) across four seasons (from 2015/16 to 2018/19; *n* = 1,985 matches). All players who participated in matches (starters and non-starters) were included. Goalkeepers and players who played less than 10 minutes during the matches were excluded because it was observed that average values obtained from these players were higher than the team average [26]. Thus, 4,252 recordings were excluded due to inclusion criteria, issues related to repeated signal loss by the system, or adverse weather conditions during the match that hindered accurate data collection. Data were provided to the authors by LaLiga, and the study received ethical approval from the University of Extremadura; Vice-Rectorate of Research, Transfer and Innovation – Delegation of the Bioethics and Biosafety Commission (Protocol number: 239/2019).

### Variables

**Players’ age.** Two variables were used to analyse this construct from two different perspectives: 1) Players’ age group: according to previous studies [13, 14], soccer players were divided into 3 age groups: young (18–24 years old), middle-aged (25–30 years old), and seniors (31–41 years old); 2) Centred players’ age: a continuous variable was generated to explore the longitudinal age effects across the four seasons (i.e., age group-mean centred; the players’ age in each season was centred to the players’ age group mean in each season).

**Match running performance**. The relative distance covered by the teams was analysed by Mediacoach at different speed ranges [23, 27]: distance covered between 21 and 24 km · h^−1^ (i.e., HIRD/min = high-intensity running distance per minute) and distance covered at more than 24 km · h^−1^ (i.e., VHIRD/min = very high-intensity running distance per minute). The relative TD variable corresponds to the summation of all the distances covered by the players per minute. Likewise, the number of high-intensity efforts performed per minute was also divided into two speed ranges: the number of very high-intensity running efforts between 21 and 24 km · h^−1^ (i.e., Sp21) and the number of sprints at speeds above 24 km · h^−1^ (i.e., Sp24). Absolute values of match running performance variables were normalized to relative values per unit of time to consider the possible differences in the total playing time of soccer matches. All the efforts that implied a minimum movement of one metre, which was maintained for a 1-second minimum, were recorded. Any recording at a speed of over 80% of the value of that category (i.e., > 24 km · h^−1^) was considered as a single register.

These data were obtained using an optical tracking system (ChyronHego; TRACAB, New York, US). This multi-camera tracking system consists of 8 super 4K high dynamic range cameras based on a positioning system (Tracab – ChyronHego VTS) that film from several angles and analyse X and Y positions of each player, thus providing real-time three-dimensional tracking. This instrument is also based on the correction of the semi-automatic VTS (the manual part of the process). The validity and reliability of the Tracab video-tracking system have been analysed, reporting average measurement errors of 2% for the total TD [2, 28]. In addition, recent studies have previously tested the agreement between the Mediacoach system and GPS devices [27]. Specifically, the magnitude of the intraclass correlation coefficients (ICC) was higher than .90.

**Season phases**. All matches of each round of the four seasons were recorded. Similar to previous research [25, 29], every season was split into four blocks of matches. Specifically, according to the number of matches per season, every season was split into four phases: matches 1–10 (i.e., Phase 1 = P1), matches 11–19 (i.e., Phase 2 = P2), matches 20–29 (i.e., Phase 3 = P3), and matches 30–38 (i.e., Phase 4 = P4).

### Statistical analysis

All statistical analyses were performed using R-studio [30]. Taking into account the characteristics of the sample, organized hierarchically, nested in groups, and with a longitudinal structure, we considered that the best procedure to analyse the data is through linear mixed models (LMM).

LMM have demonstrated their ability to cope with unbalanced and repeated-measures data [31]. For instance, variables about distances covered in matches are nested into players (i.e., each player has a record for every match they have participated in, and each match has observations of several players). Players, in turn, are also nested into different teams every season. Thus, the cross-classified multilevel models are suitable for data structures that are not purely hierarchical. Consequently, a general multilevel-modelling strategy was applied where fixed and random effects in different steps were included [31].

First, unconditional models were analysed exclusively including dependent variables (i.e., distance covered variables), to determine whether the grouping variables at Levels 2 and 3 (i.e., Players and Teams) significantly affect the intercept of each dependent variable (intercepts represent the estimated mean of each variable). Only at Level 2 (players) did relevant values of variability appear, verified from the intraclass correlation coefficient (ICC) with scores greater than 10% [31]. Subsequently, different models were performed for each of the dependent variables, introducing as fixed effects the players’ age, firstly centred for each player, and later age group and season phases. Following the procedure proposed by Heck and Thomas [31], models with different random effects (intercepts and slope) were created for each variable. Model comparison for each step was done using the Akaike information criterion (AIC; [32]) and a chi-square likelihood ratio test [33], where a lower value represented a better fitting model.

Final models, presented in Tables 2 and 3, were chosen according to better fit values of AIC, chi-square likelihood ratio test, and the significant effect of the variables. We used maximum likelihood (ML) estimation for model comparison, and, for the final model of each distance variable, we refitted the best model using restricted maximum likelihood (REML) estimation. The intercept represents the predicted value of the dependent variable when all the independent variables have a value of 0. The slope indicates the steepness of the regression line between dependent and independent variables (i.e., physical demands and player’s age centred). We reported marginal and conditional R^2^ metrics for each LMM to provide some measure of effect sizes [34]. The level of significance was set to *p* < .05.

## RESULTS

### Players, matches, and minutes distribution

Table 1 displays the matches’ and minutes’ mean distribution of all the players who participated in the study. A large number of individual match observations were registered in G2 (19,177). In general, the players of G1 played fewer matches and minutes than the players of G2 and G3 across the four seasons, whereas the players of G3 played more matches (except for season 15/16) and minutes than the players of G1 and G2 across the four seasons.

**TABLE 1:** Mean of number of matches and minutes played per season and group

Season	G1 (*n* = 7,399)		G2 (*n* = 19,177)		G3 (*n* = 10,397)	
	Matches	Minutes	Matches	Minutes	Matches	Minutes
15/16	13.10	58.88	21.09	71.23	20.32	73.35
16/17	14.24	61.17	19.69	71.62	20.11	74.70
17/18	12.59	59.33	19.80	72.08	20.59	75.94
18/19	14.69	64.42	21.11	72.10	21.11	74.13

*Note.*G1 = between 18–24 years old; G2 = between 25–30 years old; G3 = between 31–41 years old.

### Age differences in match running performance

Regarding match running performance, Table 2 displays the intercepts and slopes of TD, HIRD, and VHIRD across the years according to age group in different season phases. The intercept represents the estimated mean of each variable when the players’ age centred variable has a value of 0. In general, young players covered significantly greater TD, HIRD, and VHIRD than the rest of the players (*p* < .05) in all the season phases. The senior players covered the lowest values (*p <* .05). In addition, all players covered significantly less TD, HIRD, and VHIRD in P1 compared to the rest of the season phases (*p <* .05), with young players covering higher values.

**TABLE 2:** Player´s match TD, HIRD and VHIRD according to different age groups depending on season phases

Variables		TD (m/min.)						HIRD (m/min.)						VHIRD (m/min.)					
		Intercept (SE)	DG	DP	Slope (SE)	DG	DP	Intercept (SE)	DG	DP	Slope (SE)	DG	DP	Intercept (SE)	DG	DP	Slope (SE)	DG	DP
P1	G1	114.1 (.51)	bc	^23^	-.67 (.23)^**^		^34^	3.57 (.06)	bc	^234^	.00 (.04)	b		3.63 (.08)	bc	^234^	.03 (.05)	bc	
	G2	111.8 (.42)	ac	^234^	-.94 (.28)^***^		^24^	3.31 (.07)	ac	^234^	-.09 (.08)^***^	a	^234^	3.25 (.09)	ac	^234^	-.10 (.05)^***^	a	^2^
	G3	109.6 (.39)	ab	^234^	-.98 (.23)^***^		^2^	2.92 (.05)	ab	^234^	-.07 (.08)^**^			2.60 (.01)	ab	^234^	-.08 (.04)^**^	a	

P2	G1	114.7 (.51)	bc	^14^	-.95 (.23)^***^	bc	^34^	3.80 (.06)	bc	^14^	.01 (.04)			3.81 (.08)	bc	^1^	.03 (.05)		
	G2	113.2 (.47)	ac	^134^	-.45 (.18)^***^	a	^134^	3.54 (.08)	ac	^14^	-.01 (.08)		^1^	3.45 (.06)	ac	^14^	-.02 (.02)	a	^1^
	G3	111.1 (.59)	ab	^14^	-.52 (.15)^***^		^13^	3.14 (.06)	ab	^14^	-.02 (.08)			2.73 (.09)	ab	^1^	-.03 (.02)		

P3	G1	114.7 (.51)	bc	^14^	-1.75 (.23)^***^	bc	^12^	3.82 (.06)	bc	^14^	-.04 (.04)	b		3.82 (.08)	bc	^1^	.05 (.04)	bc	
	G2	112.8 (.41)	ac	^124^	-.94 (.11)^***^	a	^24^	3.51 (.08)	ac	^14^	-.02 (.08)		^1^	3.42 (.06)	ac	^1^	-.06 (.01)^**^	a	
	G3	111.3 (.59)	ab	^14^	-.88 (.15)^***^	a	^2^	3.13 (.06)	ab	^14^	-.05 (.02)^*^			2.77 (.09)	ab	^1^	-.05 (.03)^*^	a	

P4	G1	114.0 (.50)	bc	^23^	-2.08 (.23)^***^	bc	^12^	3.69 (.06)	bc	^123^	-.00 (.04)			3.83 (.08)	bc	^1^	-.04 (.04)		
	G2	112.2 (1.18)	ac	^123^	-1.21 (.11)^***^	ac	^123^	3.41 (.04)	ac	^123^	.01 (.08)	c	^1^	3.40 (.07)	ac	^12^	-.05 (.02)^*^		
	G3	110.5 (1.39)	ab	^123^	-.77 (.14)^***^	ab		3.06 (.05)	ab	^123^	-.05 (.08)^*^	b		2.75 (.09)	ab	^1^	-.04 (.02)		

*Note*. ^*^p < .05, ^**^p < .01, ^***^p < .001.

SE = Standard Error; TD/min. = Total distance covered per minute; HIRD/min. = High intensity running distance covered per minute; VHIRD/min. = Very high intensity running distance covered per minute; G1 = 18–24 years old; G2 = 25–30 years old; G3 = 31–41 years old; P1 = Season Phase 1; P2 = Season Phase 2; P3 = Season Phase 3; P4 = Season Phase 4; a = significant differences compared with G1; b = significant differences compared with G2; c = significant differences compared with G3; 1, 2, 3 and 4 = significant differences compared with P1, P2, P3 and P4, respectively.

The slopes represent the relationship between match running performance and the players’ age-centred variable (i.e., evolution of match running performance over the years). Concretely, TD significantly decreased over the years in all player age groups (*p* < .01), but this trend was greater across the seasons in young players. According to season phases, TD covered by young players in P1 decreased less than in the rest of the season phases, where the decrease was significantly greater (*p <* .001). TD covered by senior players decreased less, that is, it remained stable across season phases. In addition, HIRD significantly decreased in middle-aged (*p* < .001) and senior players (*p* < .01) in P1, and in senior players in P3 (*p* < .05) and P4 (*p* < .05). Finally, VHIRD significantly decreased in middle-aged (*p* < .001) and senior players (*p* < .01) in P1, in middle-aged (*p* < .01) and senior players (*p* < .05) in P3, and in middle-aged players (*p* < .05) in P4. We highlight that HIRD and VHIRD covered by young players did not decrease over the years, except in the end season phases.

Concerning the number of very high-intensity running efforts, Table 3 displays the intercepts and slopes of Sp21 and Sp24 performed per minute according to age groups in different season phases. In general, young players performed significantly more Sp21 and Sp24 than the rest of the players (*p* < .05) in all the season phases. The senior players performed the lowest number of Sp21 and Sp24 (*p <* .05). In addition, all players performed significantly fewer Sp21 and Sp24 in P1 compared to the rest of the season phases (*p <* .05). Regarding the slopes (i.e., evolution of the number of very high-intensity running efforts across the years), Sp21 significantly decreased in middle-aged (*p* < .01) and senior players (*p* < .05) in P1, and in senior players in P3 (*p* < .05) and P4 (*p* < .01). Finally, Sp24 significantly decreased in middle-aged (*p* < .001) and senior players (*p* < .001) in P1 and P2 (both *p* < .05), in senior players in P3 (*p* < .05), and in middle-aged (*p* < .001) and senior players (*p* < .001) in P4. We note that in young players the number of Sp21 performed did not decrease as much as that of Sp24.

**TABLE 3:** Number of Sp21 and Sp24 performed according to different age groups depending on season phases

Variables		Sp21 (nº/min.)						Sp24 (nº/min.)					
		Intercept (SE)	DG	DP	Slope	DG	DP	Intercept (SE)	DG	DP	Slope	DG	DP
P1	G1	.32 (.01)	bc	^234^	-.00 (.00)	b		.20 (.00)	bc	^234^	-.00 (.00)	b	
	G2	.29 (.01)	ac	^234^	-.01 (.00)^***^	a	^234^	.18 (.01)	ac	^234^	-.01 (.00)^***^	a	^234^
	G3	.26 (.01)	ab	^234^	-.01 (.00)^**^			.15 (.01)	ab	^234^	-.01 (.01)^***^		

P2	G1	.34 (.01)	bc	^14^	-.00 (.00)			.21 (.00)	bc	^14^	-.00 (.00)		
	G2	.31 (.01)	ac	^134^	-.00 (.00)		^1^	.19 (.01)	ac	^14^	-.00 (.00)^*^		^1^
	G3	.27 (.01)	ab	^14^	-.00 (.00)			.16 (.01)	ab	^1^	-.00 (.01)^*^		

P3	G1	.36 (.01)	bc	^14^	.00 (.00)			.21 (.00)	bc	^1^	.00 (.00)		
	G2	.29 (.01)	ac	^124^	-.00 (.00)		^1^	.19 (.01)	ac	^14^	-.01 (.00)^***^	a	^1^
	G3	.27 (.01)	ab	^14^	-.00 (.00)^*^			.16 (.01)	ab	^1^	-.00 (.01)^***^		

P4	G1	.32 (.01)	bc	^123^	-.00 (.00)			.20 (.00)	bc	^12^	-.00 (.00)	b	
	G2	.30 (.01)	ac	^123^	.00 (.00)	c	^1^	.19 (.01)	ac	^123^	-.00(.00)^***^	a	^1^
	G3	.26 (.01)	ab	^123^	-.01 (.00)^**^	b		.15 (.01)	ab	^1^	-.01 (.01)^***^		

*Note*. ^*^p < .05, ^**^p < .01, ^***^p < .001.

SE = Standard Error; Sp21/min. = Sprints at very high intensity running covered per minute; Sp24/min. = Sprints at more than 24 km × h^−1^ covered per minute; G1 = 18–24 years old; G2 = 25–30 years old; G3 = 31–41 years old; P1 = Season Phase 1; P2 = Season Phase 2; P3 = Season Phase 3; P4 = Season Phase 4; a = significant differences compared with G1; b = significant differences compared with G2; c = significant differences compared with G3; 1, 2, 3 and 4 = significant differences compared with P1, P2, P3 and P4, respectively.

Finally, an overview of match movement profiles according to different age groups depending on season phases is presented in Table 4. In general, young players covered greater TD, HIRD and VHIRD than the rest of the players in all the season phases. Meanwhile, the senior players covered the lowest values in TD, HIRD and VHIRD. Concerning the number of very high-intensity running efforts, young players performed more Sp21 and Sp24 than the rest of the players in all the season phases, whereas senior players performed the lowest number of Sp21 and Sp24. In addition, match running performance (i.e., TD, HIRD, VHIRD, SP21 and SP24) was lower in P1 compared to the rest of the season phases. On the other hand, match movement profiles considering the time (in minutes) played by soccer players were also analysed (See Supplementary Table).

**TABLE 4:** Match movement profiles (in meters) according to different age groups depending on season phases

Season phases	Age group	TD (m)	HIRD (m)	VHIRD (m)	Sp21 (nº)	Sp24 (nº)
		Intercept (SE)	Intercept (SE)	Intercept (SE)	Intercept (SE)	Intercept (SE)
P1	G1	10,386 (912)	285.94 (100.07)	298.15 (154.60)	25.22 (8.20)	16.08 (7.10)
	G2	10,373 (929)	271.48 (102.33)	259.93 (142.35)	23.95 (8.45)	14.53 (7.11)
	G3	10,065 (921)	258.31 (99.66)	234.44 (127.76)	22.67 (8.28)	13.31 (6.40)

P2	G1	10,535 (939)	304.78 (98.35)	315.65 (157.35)	26.81 (8.23)	17.15 (7.54)
	G2	10,484 (887)	294.73 (107.55)	282.82 (151.11)	25.88 (8.93)	15.76 (7.46)
	G3	10,201 (951)	280.37 (99.25)	247.75 (129.15)	24.49 (8.17)	14.12 (6.54)

P3	G1	10,540 (927)	310.33 (104.19)	318.67 (168.41)	27.31 (8.55)	17.18 (7.69)
	G2	10,469 (899)	291.01 (107.03)	284.30 (147.52)	25.62 (8.80)	15.74 (7.24)
	G3	10,168 (944)	272.62 (100.89)	250.92 (135.28)	23.83 (8.30)	14.08 (6.69)

P4	G1	10,425 (922)	295.59 (99.83)	314.36 (156.21)	26.03 (8.26)	16.82 (7.19)
	G2	10,419 (922)	286.66 (106.61)	280.11 (148.85)	25.22 (8.84)	15.53 (7.40)
	G3	10,162 (948)	266.48 (101.36)	243.15 (131.05)	23.26 (8.32)	13.70 (6.53)

*Note*. SE = Standard Error; TD = Total distance; HIRD = High intensity running distance; VHIRD = Very high intensity running distance; Sp21 = Number of sprints at very high intensity running; Sp24 = Number of sprints at more than 24 km × h^−1^; G1 = 18–24 years old; G2 = 25–30 years old; G3 = 31–41 years old; P1 = Season Phase 1; P2 = Season Phase 2; P3 = Season Phase 3; P4 = Season Phase 4.

## DISCUSSION

The main objective of the present study is to explore the evolution of match running performance through-out the season depending on players’ age, including, as a novel approach, a longitudinal follow-up during four consecutive seasons. The main findings of the study revealed that TD significantly decreased over the years in all the players’ age groups, although the number of high-intensity efforts performed by young soccer players remained stable and increased compared to the rest of the players. Also, match running performance (distances covered and number of high-intensity efforts performed per minute) in P1 was lower compared to the rest of the season phases.

Concerning the intercepts, our results showed that younger soccer players covered significantly greater TD, HIRD, and VHIRD and performed a significantly greater number of Sp21 and Sp24 than the other two groups (i.e., middle-aged and senior soccer players) across all season phases. These results are consistent with those previously reported in the first German league, where professional soccer players aged > 30 years showed a significantly lower performance in the TD covered, distance covered at high intensity, and the number of sprints compared with younger players [18]. Similar results were also found in the First Spanish Division. Rey et al. [13] reported that professional soccer players aged > 30 years covered significantly lower TD, distances covered at medium and high-intensity, maximum running speed, and number of high-intensity efforts compared to younger soccer players. Specifically, in elite soccer players the TD, distances covered at high intensity and number of high-intensity efforts significantly decrease each year that they get older [16]. Although acceleration and deceleration match performance were not analysed in this study, Lorenzo-Martínez et al. [15] also found that soccer players aged 31–38 years who competed in the First Spanish Division performed a significantly lower total number of accelerations and decelerations in comparison with younger players.

Regarding how the different age group players run, our results revealed that the TD covered by young soccer players (< 24 y.o.) was higher than the rest of the soccer players and it remained stable throughout the different season phases (i.e., P2, P3, and P4), whereas the TD covered by the two older age groups was lower and their TD increased as the season progressed. A possible explanation may be the fact that young soccer players played fewer matches and minutes (see Table 1) than older soccer players and covered more distance at high intensity than the rest of the players, who were more experienced in pacing their efforts [34]. When the intensity of distance covered was analysed in depth, our findings showed that young players covered greater distances at a high intensity (HIRD and VHIRD) in all the season phases (i.e., P2, P3, and P4) compared to the early season (i.e., P1). These young players are not usually part of the starter team and often play in the last part of the matches, when other players are under match-derived fatigue conditions. This situation allows young players to cover greater relative distances per minute at all running intensities than the older players, who have been forced to dosage their effort for more time during the match [35, 36].

Focusing on the evolution of match running performance throughout the season, this study showed that TD, HIRD, and VHIRD, as well as the number of high-intensity efforts, were greater during the middle season compared to the rest of season phases. Research has explored physical performance in other European leagues, reaching similar conclusions. Two longitudinal studies from the Italian Serie-A reported increases in TD and distances covered at high velocities (HIRD and VHIRD) at the end season [37, 38]. Another longitudinal study (from 2014 to 2017) from the German Bundesliga indicated across-season (P1–P4) reductions in TD covered at low intensity (below 11 km · h^−1^) but increases in distances covered at higher intensities toward the end season (P4–P6; [19]). Similar results were presented by Ponce-Bordon et al. [29], who found that soccer players covered significantly greater distances and completed a higher number of sprints in mid season. This increase in the players’ capacity of effort (greater TD covered and at higher intensities) as the season progresses may be related to the improvement of the players’ performance due to the specific adaptations derived from training and competitions [25, 39]. Conversely, a recent study of the English Championship (EC, Second Division) identified the opposite pattern of results, showing decreases in HIRD and VHIRD and the number of sprints, over the four season phases [40]. It should be noted that the aforementioned studies proposed a longitudinal analysis of the evolution of the match running performance indicators across the season without considering the players’ age. Interestingly, our results showed a reduction in the TD covered in the young players’ group as the season progresses. At the end season, the higher pressure demands to win may imply that younger players with less experience play fewer minutes as starters but relatively more at the end of the match and, therefore, with higher intensity per minute in comparison to the groups of older players [35].

One of the more significant findings to emerge from this study is the longitudinal changes in match running performance in all the four seasons analysed. It is noteworthy that taking the early season as a reference (i.e., Phase 1 of 2015/16 season), the TD showed a decrease throughout all the seasons, similar to the results of a previous study which analysed the Spanish LaLiga Santander across 8 seasons [11]. Concretely, young soccer players covered fewer metres per minute each season, whereas this trend was lower in the older age groups. Indeed, considering the following season phases, the trend became more negative, showing that, over the years, young players covered less distance as the season progressed. Taken together, these results suggest that young players across the season (from P1 to P4) and over the seasons (from 2015/16 to 18/19) could optimize their physical performance by covering less distance at lower intensities but more distance at higher velocities. This fact may be facilitated by a greater game understanding as they mature as professional players [18]. Similarly, in middle-aged and older players the TD covered significantly decreased as the season progressed but the effect of the age group throughout the four seasons was significantly lower than in younger players. That is, older players maintain their physical performance throughout the season and over various consecutive seasons. This pattern of more stable physical performance throughout the years in older players could be explained by their greater expertise, which would entail greater self-knowledge of their physical, technical, and tactical capacities, as well as their ability to apply them in the best way due to their better knowledge of the game and its demands [18]. Finally, and regarding the differences in distance covered at high intensity, the effect of age is completely different, showing greater distance covered at high intensity over the seasons in younger players and less distance covered at high intensity (HIRD and VHIRD) over the seasons in older players. Lorenzo-Martínez et al. [14] found that younger players were more stable, maintaining the ability to perform high-intensity running and sprinting in comparison with older players. Our findings have shown that older players tend to reduce their ability to repeat high-intensity efforts over the years, due to different factors, such as their age-related decrease in strength [41] or diminishing soccer-specific physical abilities [16].

### Limitations and Future Directions

The present study has several limitations that should be considered. Firstly, future studies on match running performance should be analysed according to players’ playing position and the type of participation in the match (starters vs. non-starters, entire game, 1^st^ or 2^nd^ half, last quarter of the match, etc.), given that younger players may participate more in the last minutes of the match, and this could modify the profile of their efforts and intensities of running. Secondly, this study did not consider some contextual-related variables, such as the opponent quality, game model, and fixture congestion, which may influence players’ match running performance.

## CONCLUSIONS

The current study represents the first longitudinal research to explore the evolution of match running performance depending on the age of soccer players who competed in four consecutive seasons. Firstly, young soccer players covered significantly greater distances at all intensities than the older groups, so these players showed a better physical performance independently of the season phase (P1–P4). Secondly, young players increased distances at a high intensity as the season progressed. This age group plays fewer matches and minutes, so this situation makes it easier for younger players to perform greater relative distances per minute at all running intensities than older players. Finally, in middle-aged and older players the distances covered at high intensity significantly decreased across the seasons. Therefore, soccer coaches should take into consideration that the evolution of match running performance over the season is especially influenced by the player’s age. This should be considered when adjusting competition minutes, selecting the distribution of players depending on the anticipated match physical demands, and especially when adapting physical training regimes to the evolution of players’ match running performance.

### Practical applications

This study provides very useful knowledge to soccer coaches when adjusting competition minutes, selecting the distribution of players depending on the anticipated match physical demands. Therefore, coaches could select younger soccer players at the early season due to better physical performance. Moreover, match running performance significantly changes in the different phases of the season and, more importantly, these changes are highly influenced by the players’ age. Therefore, our results could help strength and conditioning coaches, especially when adapting physical training regimes to the evolution of players’ match running performance according to the season phases to avoid overuse and optimize their physical performance.

## Figures and Tables

**Table S1:** Match movement profiles (in meters) according to different age groups depending on season phases considering time played by soccer players

Season phases	Age group	TD (m)	HIRD (m)	VHIRD (m)	Sp21 (nº)	Sp24 (nº)
		Intercept (SE)	Intercept (SE)	Intercept (SE)	Intercept (SE)	Intercept (SE)
P1	G1	7,614 (3,289)	222.97 (114.48)	231.55 (150.65)	19.74 (9.80)	12.57 (7.36)
	G2	8,471 (2,976)	234.22 (111.87)	227.24 (141.18)	20.62 (9.49)	12.65 (7.24)
	G3	8,438 (2,747)	223.05 (107.89)	202.00 (127.28)	19.57 (9.14)	11.47 (6.60)

P2	G1	7,908 (3,339)	243.23 (119.95)	251.57 (158.70)	21.40 (10.32)	13.69 (7.92)
	G2	8,628 (3,005)	253.98 (120.43)	244.50 (153.30)	22.25 (10.24)	13.57 (7.76)
	G3	8,697 (2,714)	245.89 (112.87)	218.77 (131.33)	21.43 (9.51)	12.45 (6.87)

P3	G1	7,822 (3,409)	242.76 (124.38)	248.29 (166.63)	21.34 (10.66)	13.42 (8.17)
	G2	8,634 (2,953)	253.33 (118.86)	245.97 (148.55)	22.24 (10.09)	13.62 (7.54)
	G3	8,493 (2,792)	236.89 (113.16)	215.02 (135.57)	20.65 (9.50)	12.08 (6.92)

P4	G1	7,887 (3,290)	236.94 (118.37)	248.96 (156.07)	20.81 (10.11)	13.29 (7.62)
	G2	8,575 (2,938)	246.37 (117.35)	240.93 (148.14)	21.59 (9.94)	13.31 (7.56)
	G3	8,405 (2,898)	229.30 (112.19)	209.31 (132.56)	19.96 (9.45)	11.77 (6.83)

*Note*. The time (in minutes) played by soccer players was considered for this analysis. SE = Standard Error; TD = Total distance; HIRD = High intensity running distance; VHIRD = Very high intensity running distance; Sp21 = Number of sprints at very high intensity running; Sp24 = Number of sprints at more than 24 km × h^−1^; G1 = 18–24 years old; G2 = 25–30 years old; G3 = 31–41 years old; P1 = Season Phase 1; P2 = Season Phase 2; P3 = Season Phase 3; P4 = Season Phase 4.
